# A Stepped Care Model of Patient Navigation to Enhance Engagement with Perinatal Mental Health Care

**DOI:** 10.1089/heq.2020.0077

**Published:** 2020-11-13

**Authors:** Christina Bricca DiSanza, Pamela A. Geller, Alexa Bonacquisti, Bobbie Posmontier, June Andrews Horowitz, Lisa A. Chiarello

**Affiliations:** ^1^ Member and Client Services, Progyny, Inc., New York, New York, USA.; ^2^ Department of Psychology, Drexel University, Philadelphia, Pennsylvania, USA.; ^3^ Department of Graduate Counseling Psychology, Holy Family University, Philadelphia, Pennsylvania, USA.; ^4^ Jefferson College of Nursing, Thomas Jefferson University, Philadelphia, Pennsylvania, USA.; ^5^ College of Nursing and Health Science, University of Massachusetts Dartmouth, Dartmouth, Massachusetts, USA.; ^6^ Department of Physical Therapy and Rehabilitation Sciences, Drexel University, Philadelphia, Pennsylvania, USA.

**Keywords:** postpartum depression, patient navigation, perinatal mental health, stepped care

## Abstract

During the perinatal period, women are at increased risk for developing perinatal mood and anxiety disorders (PMADs). As perinatal mental health screening efforts increase, significantly more women will be identified who require mental health services. Evidence-based treatments exist, yet many women do not receive adequate care. Patient navigation (PN) offers a promising patient-centered approach to improve treatment attendance and engagement. The purpose of this study is to describe the development of a stepped care PN service at an intensive outpatient program for women with PMADs. Our experience incorporating this model of PN revealed significant features that may guide other treatment care facilities to adopt this service to increase identification and connection to care.

## Introduction

### Background on perinatal mood and anxiety disorders

Perinatal mood and anxiety disorders (PMADs) are the most common mental health conditions for women of reproductive age^1^ and disproportionately affect women of color and those with low-income status.^2^ If untreated, PMADs can have significant adverse outcomes for women and their children,^3^ including preterm birth^4^ and maternal suicide.^5^ Long-term effects of PMADs include potential impairments in infant cognitive and social-emotional development and infant behavior. In addition to adverse effects on the infant, poor family functioning may also be a long-term result of PMADs.^6,7^

Despite increased awareness and screening for PMADs^8^ and availability of effective evidence-based psychotherapies,^9–11^ many women do not receive treatment for their distress.^12^ Perinatal mental health treatment uptake and maintenance are influenced by sociodemographic factors; attitudes and values toward providers and mental health services; knowledge about PMADs and available treatments; severity of symptoms; a woman's perceived or evaluated need for treatment; societal and cultural norms; insurance status; and childcare availability.^13^ In response to the complexity of accessing perinatal mental health care, patient navigation (PN) services offer a promising patient-centered approach to promote treatment attendance, engagement, and retention among patient populations with varied health and social needs. Unlike case management, PN is delivered by a layperson or unlicensed worker focused on eliminating barriers, not providing clinical care.^14,15^

### Purpose

The purpose of this study is to describe the development of a PN service at *Mother Baby Connections* (MBC), an intensive outpatient program for women with PMADs at Drexel University in Philadelphia, PA.^16^ This study describes the role of patient navigator at MBC and introduces a stepped care model of PN services for perinatal mental health care that matches the intensity of PN to patients' needs based on PMADs symptom severity, health literacy, social support, and socioeconomic resources. Our experience in utilizing a patient navigator for women seeking treatment for PMADs may guide other programs in adopting this kind of service. Institutional Review Board approval was obtained for any research occurring at the Mother Baby Connections program; however, no patient data are reported in this paper.

## Patient Navigator Responsibilities in Perinatal Mental Health Care

The primary responsibilities of the patient navigator at MBC are to coordinate care, optimize treatment attendance, and enhance engagement by minimizing barriers to treatment. For an overview of these distinct but interrelated responsibilities that make up the role of patient navigator at MBC, see Table 1. At MBC, the position of the patient navigator has been filled by graduate students from related disciplines (e.g., psychology and nursing) and advanced undergraduate students with training in PMADs, cultural humility,^17^ risk assessment, and motivational interviewing skills to enhance patient readiness for treatment.^18^

**Table 1.** *Mother Baby Connections* Patient Navigation Model: Primary Roles and Responsibilities

Role	Responsibilities
Coordinate care	Locate and discuss community resources
	Coordinate referrals for external providers
	Text messaging to improve communication
	Provide relevant health care information at an eighth grade reading level
	Coordinate administration of assessment measures every 4 weeks
Optimize treatment attendance	Identify and minimize barriers
	Provide information to enhance transportation to/from clinic
	Empathy to normalize challenges of PMADs
	Bring patient access problems to the team
Support treatment engagement	Inquire about patients' experience
	Validate decision-making and communication with team
	Address small organizational tasks (e.g., reminders)
	Encourage practice of learned coping strategies (e.g., deep breathing)
	Promote question-asking and acquisition of health knowledge

PMADs, perinatal mood and anxiety disorders.

### Coordinate care

The navigator at MBC uses text message and phone communication to provide appointment scheduling and care coordination, deliver weekly and daily appointment reminders, build rapport, relay emotional support to patients, and offer referrals as needed. This communication approach is consistent with extant findings that suggest a significant positive association between the number of text messages and likelihood for follow-up attendance among perinatal women who received PN services.^19^ To address low health literacy among some perinatal women, the navigator provides relevant health care information at an eighth grade reading level. The navigator also coordinates administration of assessment measures every 4 weeks, including the Edinburgh Postnatal Depression Scale,^20^ and communicates patient response to the MBC treatment team in weekly care coordination meetings.

### Optimize treatment attendance

To optimize treatment attendance, the patient navigator identifies and minimizes barriers. Given that stigma is a significant barrier to mental health treatment, the navigator uses empathy to normalize challenges during the perinatal period and reinforce nonjudgmental support from the treatment team. The patient navigator also enhances patients' access to care and resources outside of MBC through individualized referral management and follow-up. For patients with transportation challenges, the navigator provides information about public transportation, discounted parking, ride-share services, and transportation assistance. At discharge, the navigator facilitates treatment referrals and provides information about parenting resources such as parent–baby groups in the community. Throughout treatment and immediately postdischarge, the navigator tracks all referrals and engages in follow-up to ensure that patients' needs are being addressed. The navigator discusses patient access problems with the team to promote patient engagement with treatment, attendance, and retention.

### Promote engagement with treatment

Low patient engagement in health- and self-care may result in a self-perpetuating cycle of distress and ongoing symptoms.^21^ To promote patient engagement with their care, the navigator educates patients on program structure, treatment modalities, and expectations. In addition, the navigator asks the patient about her program experience to elicit conversations about decision-making in care and patient–provider interactions. The navigator collaborates with patients to identify strategies to stay engaged with treatment and reach health goals (e.g., setting phone reminders). Using information shared in care coordination meetings to reduce patient distress, the navigator reinforces patient adoption of coping strategies learned with therapists during MBC sessions (e.g., diaphragmatic breathing) when appropriate.

### Stepped care model of PN at MBC

**FIG. 1.**
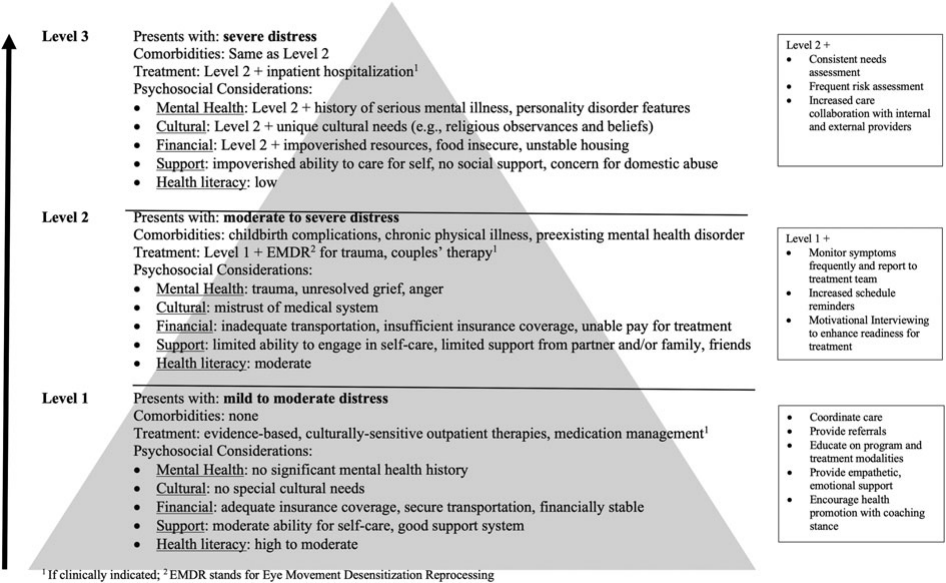
Stepped care model of patient navigation for women with perinatal mood and anxiety disorders at *Mother Baby Connections.* EMDR, eye movement desensitization reprocessing.

To promote treatment access and engagement of patients with varying levels of distress and barriers to entry, the navigator utilizes a stepped care approach to match intensity of PN services to patient needs (Fig. 1). To assess the intensity of the PN service needed throughout treatment, the navigator considers the patient's level of support, logistical and emotional barriers, and psychosocial concerns in collaboration with the MBC treatment team. Patients with increased distress and greater barriers to engagement (e.g., inconsistent levels of support, low health literacy, and limited income) signal the need for a higher intensity PN intervention.

The stepped care model accommodates the diversity of women who seek treatment for PMADs with three levels of PN services. The navigator deploys Level 1 PN services for women presenting with mild to moderate distress, high health literacy, financial stability, and moderate support. Level 1 PN services include scheduling/care coordination, identifying needs and providing resources, educating patients about program and treatment modalities, and offering emotional support as indicated. The navigator steps up PN services to Level 2 for women presenting with moderate to severe distress, clinical comorbidities, increased treatment needs, and limited financial and social support. Level 2 PN services include all services in Level 1 plus frequent symptom monitoring and reporting to treatment team, increased schedule reminders, and elements of motivational interviewing to enhance readiness for treatment.^17^ To meet the needs of women with severe distress (e.g., recent hospitalization due to PMADs, impoverished support or resources, and poor self-care), the navigator deploys Level 3 PN services that include all services in Level 2 plus consistent needs assessment, frequent risk assessment and coordination with clinical team, and increased care collaboration with internal and external providers. Through the stepped care approach, the navigator provides more intensive coordination and support throughout treatment for patients who are at-risk for poor mental health outcomes.

## Limitations and Future Directions

Although we have provided a description of our stepped care PN model for perinatal mental health programs, this model currently lacks formal empirical investigation. Despite this, a recently published description of MBC highlights the positive patient outcomes regarding both mental health symptoms and patient satisfaction for the MBC program overall.^16^ However, outcomes cannot be attributed to PN services specifically. Thus, program evaluation research is necessary to assess the specific gains and aspects of patient satisfaction that can be attributed to PN services.

Among perinatal mental health programs in the United States surveyed in 2017, more than a quarter had PN services^22^; however, the impact of PN services on engagement in mental health programs has not yet been formally investigated. Through regular documentation, PN interactions with patients may be tracked and coded by specific PN service provided, intervention intensity, barriers addressed, and referrals provided. This information could then be utilized in formal analyses examining the relationship between stepped care PN services and impact on attendance, engagement with treatment, and outcomes (e.g., PMADs symptoms and program satisfaction). Future systematic evaluations may consider the utility of PN services to increase patient engagement in specialized outpatient PMADs care and decrease potentially preventable hospitalizations. Further research is also needed to demonstrate the viability and potential impact of PN in a variety of settings where women with PMADs may receive services, such as inpatient psychiatric units, integrated primary care or obstetrics and gynecology offices, and community clinics.

## Conclusion

To enhance treatment access and engagement particularly for perinatal women with substantial barriers to treatment entry and retention, MBC utilizes a patient navigator to coordinate care. To improve treatment attendance, the navigator delivers frequent and supportive text messaging communication, streamlined scheduling, and care coordination, and provides comprehensive resource and referral management. The navigator promotes patient engagement in care by providing support and encouraging small behavior changes to support health goals, in conjunction with the treatment team. PN services are “stepped up” to match patient needs with an appropriate intensity of PN intervention, such as delivering ongoing needs assessment, providing referrals and increasing coordination with internal and external providers.

Integrating PN services into perinatal mental health programs has the potential to improve treatment attendance and outcomes particularly among women with heightened distress and/or increased barriers to care. In the future, evaluation of the content and dose of perinatal mental health PN services would be helpful to characterize the approach and to best understand how to achieve optimal outcomes for this specific population. As such, PN services, fashioned after the stepped care model piloted at MBC, may improve engagement and retention of women in treatment for PMADs with varying levels of distress, resources, and clinical needs.

## Abbreviations Used

EMDR: eye movement desensitization reprocessing
MBC: *Mother Baby Connections*
PMADs: perinatal mood and anxiety disorders
PN: patient navigation
